# Influence of extraction techniques on antioxidant properties and bioactive compounds of loquat fruit (*Eriobotrya japonica* Lindl.) skin and pulp extracts

**DOI:** 10.1002/fsn3.201

**Published:** 2015-01-21

**Authors:** Mojtaba Delfanian, Reza Esmaeilzadeh Kenari, Mohammad Ali Sahari

**Affiliations:** 1 Department of Food Science and Technology, Sari Agriculture and Natural Resources UniversitySari, Mazandaran, Iran; 2 Department of Food Science and Technology, Faculty of Agriculture, Tarbiat Modares UniversityTehran, Iran

**Keywords:** Antioxidant activity, *Eriobotrya japonica*, solvent extraction, ultrasound-assisted

## Abstract

In this study, the bioactive compounds of loquat fruit (*Eriobotrya japonica* Lindl.) skin and pulp extracted by two extraction methods (solvent and ultrasound-assisted) with three solvents (ethanol, water and ethanol–water) were compared to supercritical fluid extraction. The antioxidant activities of skin and pulp extracts were evaluated and compared to tertiary butylhydroquinone (TBHQ) using 2, 2-diphenyl-1-picrylhydrazyl (DPPH˙) radical scavenging, *β*-carotene bleaching, and the Rancimat assays. In DPPH assay solvent extracts of skin by ethanol (SSE) and ethanol–water (SSEW) showed strong inhibitory activity. The SSEW also showed the highest inhibition percentage of 85.58% by the *β*-carotene bleaching assay and longest induction time of 4.78 h by the Rancimat method. The large amount of tocopherols and phenolics contained in the skin extract may cause its strong antioxidant ability. The results indicated that the solvent extraction with ethanol–water produced the maximum extraction yield of phenolic and tocopherol compounds from loquat fruit skin and pulp. Furthermore, solvent extraction was the most effective in antioxidant activity of the extracts compared to other extraction techniques.

## Introduction

To prevent oxidation of oils and fats, the use of antioxidants is mandatory because compounds produced from the oxidation of oil, such as hydroperoxide, hydroxyl radical, and a single oxygen capacity can damage biological molecules (Mohdaly et al. 2010). This causes cellular damage and the development of physiological abnormalities such as premature aging, neurological, and heart disease (Suja et al. 2004).

Therefore, synthetic antioxidants such as butylated hydroxytoluene (BHT), butylated hydroxyanisole (BHA), and tertiary butylhydroquinone (TBHQ) are often used to increase the oxidative stability of oils. Studies have shown that these antioxidants are toxic and carcinogenic. Therefore, the use of natural antioxidants is required as alternatives to synthetic antioxidants (Zhang et al. 2010). Various studies have shown plants to be a rich source of natural antioxidants. Compounds with antioxidant properties found in plants include the vitamins A, E, and phenolic compounds, flavonoids, tannins, and lignins (Razali et al. 2012).

Loquat (*Eriobotrya japonica*) is an Asian fruit in the family of Rosaceae. The species is native to southeastern China and mainly grow in subtropical and mild temperate regions (Cha et al. 2011). Currently, it is also cultivated in other areas, namely in South Africa, South America, Australia, and California. Loquat is an evergreen tree with short branches that flower in late autumn or early winter (Hong et al. 2008). Its fruits ripen in late winter or early spring. Loquat is a round yellow fruit with a smooth and downy form that has a white pulp which has three to five brown seeds. Its sweet or sour taste depends on the area where it grows (Ercisli et al. 2012). Loquat fruit has good antioxidant properties due to the presence of phenolic (benzoic acid and hydroxy cinnamic derivatives) and tocopherol compounds (Tosun et al. 2009).

Nowadays, various techniques such as ultrasound-assisted and supercritical fluid extraction are used to extract bioactive compounds from plants. Ultrasonic waves with frequency of more than 20 KHz are in two forms of the probes and bath. Ultrasound has high ability to extract phenolic compounds and that is the reason it is being used frequently (Shirsath et al. 2012). Ultrasound cavitation effect can cause damage to the cell wall and thus improve the extraction yield. Sometimes ultrasound waves may possibly destroy the antioxidant compounds and reduce the extraction yield (Cao et al. 2009). Thus, implementation of this method for any plant material requires further investigation.

The supercritical fluids were also used for the extraction of antioxidant compounds. Supercritical state was achieved when the temperature and the pressure of a substance rose over its critical value, in which the fluid has the characteristics of both liquids and gases (Wang and Weller 2006). Selection of supercritical fluids is important to develop a supercritical fluid extraction method. Mostly, supercritical CO_2_ is used because it is inexpensive, nonflammable and nontoxic (Martinez-Correa et al. 2011). Supercritical CO_2_ is a suitable solvent for the extraction of nonpolar compounds such as hydrocarbons. Many polar compounds such as phenols, tocopherols, glycosides and alcohols have poor solubility in supercritical CO_2_ (Hamburger et al. 2004). The use of modifier solvents such as ethanol, acetone, and methanol can effectively increase the solubility and extraction of polar compounds. Although methanol has greater ability to extract polar compounds, but due to its toxicity ethanol is used more frequently (Lang and Wai 2001). Recently this method has been used extensively because there are various nonpolar compounds in plants which have relatively good antioxidant effects.

Solvent extraction method is a traditional method for extraction and is more frequently used for the isolation of bioactive compounds. In this method, extraction yield of bioactive compounds is dependent on conditions of extraction and the solvent polarity. Since there was no study reporting any kind of damaging effect to plant bioactive compounds in the solvent extraction method, hence it is being used extensively for extraction, but the long process time in this method caused scientists to search for an alternative method (Rodríguez-Rojo et al. 2012).

Therefore, the aim of this work was to compare the effects of solvent extraction and ultrasound-assisted techniques on antioxidant activity of loquat fruit skin and pulp extracts with supercritical CO_2_ extraction.

## Materials and Methods

### Materials

Loquat fruits were collected from fields in Sari in the Mazandaran province, Iran. All reagents used in the experiments were of analytical grade and obtained mostly from Sigma Chemical Co. (St. Louis, MO). Solvents used for the extraction of plant samples were purchased from Merck (Darmstadt, Germany).

## Extraction Methods

### Solvent extraction

The skin and pulp of the loquat fruit were separated and dried in the shade in natural conditions. Dried powder of the samples (20 g) were mixed with 100 ml of each solvent (ethanol, water, ethanol–water (1:1)). The mixture was stirred in a shaker (LABTRON Ls-100) at 160 rpm away from light at room temperature for 48 h. The extracts were filtered and solvents evaporated using a rotary evaporator (Heidolph, Schwabach, Germany) at 50°C. The extracts were stored at −20°C until testing (Tachakittirungrod et al. 2007).

### Ultrasound-assisted extraction

Dried powders of samples (20 g) were mixed with 100 mL of each solvent (ethanol, water, ethanol-water (1:1)). All the samples were placed in dark brown-colored reagent bottles with narrow necks and mixed for 30 min in an ultrasonic water bath (KQ-250DB; Kunshan Ultrasound Co. Ltd., Elma, model 690/H, Cottbus, Germany) of 35°C at 35 KHz (100% power). The extracts were filtered and solvents evaporated using a rotary evaporator. The concentrated extracts were stored in a freezer (Albu et al. 2004).

### Supercritical CO_2_ extraction

A Suprex MPS/225 Multipurpose system (Roth Scientic, Basingstoke, Hampshire, U.K.) was used for the extraction of bioactive compounds. In this method, extractions of 20 g of dried powder from the skin and pulp were accomplished with 100 mL ethanol at 35°C, 100 bar for 30 min. The extracts were filtered and concentrated using a rotary evaporator. The concentrated extracts were stored under refrigeration until further analysis (Luengthanaphol et al. 2004).

### Determination of extraction yield

The extraction yield (%) according to the method described by Tian et al. (2012) is calculated as follows:

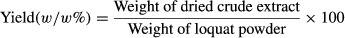


### Determination of total tocopherol content

The content of tocopherol compounds was determined according to the colorimetric method described by Wong et al. (1988). A calibration curve of pure *α*-tocopherol in toluene was performed in a concentration range of 0–240 *μ*g/mL. The extract (0.2 g) was dissolved in 5 mL of toluene and then 3.5 ml of 2, 2′-bipyridine (0.07% w/v in 95% aqueous ethanol) and 0.5 mL of FeCl_3_-6H_2_O (0.2% w/v in 95% aqueous ethanol) were added into that mixture. The solution was made up to 10 mL with 95% aqueous ethanol. After standing for 1 min, the absorption at 520 nm was determined by using a spectrophotometer (Cintra 20; GBC, Dandenong, Australia). The results are expressed as microgram of *α*-tocopherol equivalents per gram extract (*μ*g *α*-tocopherol/g).

### Determination of total phenolic content

The content of phenolic compounds in the extracts was determined according to the method of McDonald et al. (2001). Briefly, 0.5 mL of extract was mixed with 2.5 mL of 10-fold-diluted Folin–Ciocalteu reagent and 2 mL of 7.5% sodium carbonate. Then, the mixture was shaken for 1 min and allowed to stand at room temperature for 15 min. Absorbance of the solution was measured at 765 nm using a spectrophotometer (Cintra 20; GBC). The concentration of phenolic compounds was estimated using a calibration curve traced with gallic acid in methanol at concentrations of 0.04–0.4 mg/mL as a polyphenol reference. Results were expressed as *μ*g gallic acid per g extract (*μ*g GAE/g). Each test was repeated three times, and the results were averaged.

### DPPH˙ radical scavenging activity

The ability of the extracts to scavenge 2, 2-diphenyl-1-picrylhydrazyl radical (DPPH˙) was determined by the method described by Burits and Bucar (2000). Briefly, 50 *μ*L of various dilutions of the test materials (pure antioxidants or loquat extracts) were mixed with 5 mL of a 0.004% methanolic DPPH˙ solution. The reaction mixtures were shaken vigorously and incubated in the dark for 30 min. The absorbance of the solution was measured at 517 nm against a blank. The radical scavenging activity of each solution was calculated as inhibition percentage according to the following equation:

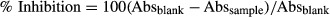


### *β*-Carotene bleaching assay

Antioxidant activities of loquat extracts were determined using the *β*-carotene bleaching method, as described by Amarowicz et al. (2010). Briefly, 5 mg of *β*-carotene were dissolved in 10 ml of chloroform and 600 *μ*l *β*-carotene solutions was mixed with 40 mg of linoleic acid and 400 mg of Tween 40 emulsifier in a round-bottom flask. Then, chloroform was removed in a rotary vacuum evaporator. Oxygenated distilled water (100 mL) was added to the flask and the resulting mixture was stirred vigorously. Five milliliters of the emulsion were transferred into tubes containing 200 *μ*L of different concentrations of extracts. After mixing, the absorbance of the samples was measured at 470 nm at initial time (*T* = 0) against a blank. The remaining samples were placed in dark for 24 h. Then, the absorbance of each sample was measured at 470 nm (Abs_24_). Antioxidant activity was calculated as inhibition percentage according to the following equation:

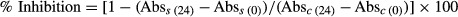
where Abs_s (24)_ is the absorbance of the antioxidant at 24 h, Abs_c (24)_ is the absorbance of the blank at 24 h, Abs_s (0)_ is the absorbance of the antioxidant at 0 times, and Abs_c (0)_ is the absorbance of the blank at 0 time.

### Rancimat analysis

A Metrohm 743 Rancimat instrument (Herisan, Switzerland) was used in the experiment. Air supply was maintained at 15 L/h and the temperature was kept at 120°C (Farhoosh and Tavassoli-Kafrani 2011).

### Statistical analysis

All experiments were conducted at three levels of measurements and results reported as mean ± standard deviation (SD). The data were analyzed by analysis of variance (ANOVA), followed by Duncan test to detect significant differences between means of treatments at *P* < 0.05. Statistical analysis was performed using SPSS statistical software (version 19; SPSS Inc., Chicago, IL).

## Results and Discussion

### Extraction yields

**Figure 1:**
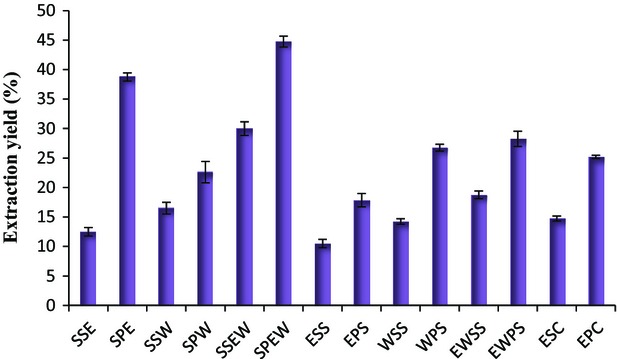
Extraction yield (%) of loquat fruit skin and pulp extracts. *Note:* Solvent extracts of skin by ethanol (SSE), water (SSW), and ethanol–water (SSEW). Solvent extracts of pulp by ethanol (SPE), water (SPW), and ethanol–water (SPEW). Ultrasound-assisted extract of ethanol (ESS), water (WSS) and ethanol–water (EWSS). Ultrasound-assisted extract of ethanol (EPS), water (WPS) and ethanol–water (EWPS). Ethanol extract of skin by supercritical CO_2_ method (ESC). Ethanol extract of pulp by supercritical CO_2_ method (EPC).

Loquat fruit skin and pulp contains considerable amounts of extractable compounds (Fig.1). The extraction yield of loquat fruit skin and pulp range from 10.5 to 30 and 17.85 to 44.75%, respectively. ANOVA results indicated that the effect of solvent polarity, extraction methods, and their interaction with extraction yield were significantly different (*P* < 0.05). The mixtures (1:1) of ethanol with water had a high ability to extract substances from the parts of the fruit. Water has great ability to extract polar compounds because its polarity is higher than other solvents. As the water is not able to extract compounds with high carbon bonds, so solvent with a suitable extraction coefficient can be prepared by mixing water and ethanol (González-Montelongo et al. 2010). The results also revealed that the solvent extraction method was more effective for extraction of compounds from the various parts of the loquat fruit compared to ultrasound-assisted and supercritical CO_2_ extraction methods. Our results were in agreement with previously published results such as the work by Plánder et al. (2012) and Sánchez-Vioque et al. (2013). The lower capability of supercritical CO_2_ for extraction of polar compounds and destruction of bioactive compounds against ultrasound waves are the reasons of high extraction yield of solvent method (Tian et al. 2012). Our results indicated under the same conditions the extraction yield of loquat pulp was significantly higher compared to that of skin. Similar results were obtained by Li et al. (2006), which reported the extraction yield in pomegranate skin extract was lower than that of pulp. Moreover, Barros et al. (2011) found the maximum extraction yield in various parts of the Brazilian citrus was in pulp extracts.

### Total tocopherols content

Tocopherols are a group of antioxidant compounds that prevent oxidation progress by scavenging peroxyl radicals without reacting in further chain-propagating steps (Mussatto et al. 2011). Table1 shows the total tocopherol compounds found in skin and pulp extracts of loquat fruit. The amount of tocopherols in loquat fruit pulp and skin extracted by different solvents and techniques ranged from 71.15 to 205.42 and 231.23 to 515.68 *μ*g *α*-tocopherol/g. Choosing the appropriate solvent is one of the most important factors in obtaining extracts with a high content of phytochemical compounds (González-Montelongo et al. 2010). Ethanol, water, and mixtures of these solvents had dissimilar efficiencies in extracting tocopherol compounds from loquat fruit skin and pulp. Water extracted the least amount of tocopherol compounds. The results indicated that the ethanol–water mixture is an effective solvent for extracting tocopherol compounds from various parts of the fruit. This result concurred with previously published results such as the work by González-Montelongo et al. (2010) which found maximum total tocopherols in the methanol–water (1:1) extracts from banana peel, whereas lower values were found for water. In general, the high amount of tocopherol compounds were found in skin extracts compared to pulp extracts under the same extraction conditions. The comparison of the extraction methods showed the solvent extraction was more effective in extraction of tocopherol compounds compared to ultrasound-assisted and supercritical CO_2_ extraction.

**Table 1:** Total phenolic and tocopherol compounds of loquat fruit skin and pulp extracts

Sample	Total phenol (*μ*g/g)	Total tocopherol (*μ*g/g)
SSE	664.53 ± 2.59c	486.53 ± 0.67b
SPE	249.96 ± 2.1i	162.34 ± 0.78j
SSW	272.51 ± 2.19g	231.23 ± 1.15f
SPW	147.39 ± 0.81m	71.15 ± 0.51n
SSEW	879.54 ± 3.91a	515.68 ± 0.39a
SPEW	387.69 ± 2.6f	205.42 ± 0.3h
ESS	394.67 ± 4.01e	388.58 ± 0.25c
EPS	209.01 ± 2.42k	187.73 ± 0.64i
WSS	264.89 ± 1.08h	309.86 ± 0.51e
WPS	164.96 ± 2.28l	107.3 ± 0.51l
EWSS	760.71 ± 3.63b	353.2 ± 0.5d
EWPS	219.40 ± 1.97j	98.5 ± 0.53m
ESC	425.02 ± 0.00d	228.41 ± 0.15g
EPC	206.45 ± 0.55k	146.36 ± 0.15k

Values (Mean ± SD, *n* = 3) in the same column with different letters are significantly different (*P* < 0.05).

*Note:* Solvent extracts of skin by ethanol (SSE), water (SSW), and ethanol–water (SSEW). Solvent extracts of pulp by ethanol (SPE), water (SPW) and ethanol–water (SPEW). Ultrasound-assisted extract of ethanol (ESS), water (WSS) and ethanol–water (EWSS). Ultrasound-assisted extract of ethanol (EPS), water (WPS) and ethanol–water (EWPS). Ethanol extract of skin by the supercritical CO_2_ method (ESC). Ethanol extract of pulp by the supercritical CO_2_ method (EPC).

### Total phenolic content

Polyphenolic compounds are widely available in plants (Wang et al. 2012) and reports show that there is a positive relation between total phenolic content and antioxidant activity in many plant species (Yasoubi et al. 2010). Phenolic compounds in plants are known as potent in vitro antioxidants due to their ability to donate hydrogen or electrons and to form stable radical intermediates (Li et al. 2006). Table1 shows the total phenolic content of the various fruit parts, which ranged from 147.39 to 387.69 (*μ*g/g) in pulp and 264.89 to 879.54 (*μ*g/g) in skin extracts. Generally, the total phenolic compound content was higher in ethanol–water extracts compared to water and ethanolic extracts of the same fruit parts. Recently, a wide variety of total phenolic content was reported in loquat fruits cultivars that ranged from 129 to 578 *μ*g GAE per g in Turkey (Polat et al. 2010) and 240 to 572 *μ*g GAE per g in China (Xu and Chen 2011). According to Rop et al. (2011) and Milivojevic et al. (2012), the phenolic content and composition of fruits depend on the genetic and environmental factors as well as postharvest processing conditions. Our results showed the total phenolic in loquat skin was higher than pulp that concurred with the results of Ferreres et al. (2009) which calculated the total phenolic content of different varieties of loquat fruit pulp that was significantly lower compared to that of skin. Similar previously published results such as the work by Luengthanaphol et al. (2004), Goli et al. (2005), and Sánchez-Vioque et al. (2013) it was revealed that the solvent extraction method was more effective to extract phenolic compounds compared to ultrasound-assisted and supercritical CO_2_ extraction methods. Based on low levels of extraction yield of phenolic and tocopherol compounds in the supercritical CO_2_ extraction method, we realized that these compounds had high polarity since CO_2_ has greater tendency to extract nonpolar compounds (Hamburger et al. 2004).

### DPPH˙ radical scavenging activity

The DPPH˙ radical scavenging is widely used as a method to determine antioxidant activity in a relatively short period compared to other methods (Li et al. 2006). Table2 shows the ability of the loquat fruit skin and pulp extracts to scavenge the DPPH˙ radical as the inhibition percentage at concentrations of 100–1000 ppm. In this assay, scavenging of free radicals increased as concentration of the extracts increased, which is due to the increasing amount of phenolic and tocopherol compounds at higher concentrations of the extracts, similar to the previously published results such as the works of Chaillou and Nazareno (2006) and Zhang et al. (2010). By increasing the concentrations of phenolic compounds, the number of hydroxyl groups available in the reaction medium increased. So, the possibility of hydrogen donation to free radicals will increase (Sánchez-Vioque et al. 2013). The extracts with the highest DPPH˙ radical-scavenging capacity were in this order: SPE at 100 ppm, SPE and ESS at 200 ppm, SPE, SSEW and ESS at 300 ppm (with no significant difference), SSEW at 400 ppm and SSE at 1000 ppm. The inhibition percentage of SSE at 1000 ppm achieved more than 66.7%, which almost matched with TBHQ. Our results clearly showed that skin samples extracted by ethanol–water (1:1) and pulp extracted by ethanol had higher antioxidant activity in different methods. In addition, from these results, we found that antioxidant activity of pulp extracts were less than the skin extracts. This conclusion was confirmed by the results of previously published results such as the works of Koba et al. (2007) and Ferreres et al. (2009), which showed that skin had higher levels of antioxidant compounds compared to the pulp. Also, Silva et al. (2004) and Omena et al. (2012) reported the antioxidant potential of quince and the exotic Brazilian fruit was higher in skin than pulp. Consequently, we found that the samples extracted by the solvent extraction method have better performance against DPPH˙ radical in comparison with ultrasound-assisted and supercritical CO_2_ extraction.

**Table 2:** Comparison of the capacity of loquat fruit skin and pulp extracts to scavenge DPPH˙ radicals.

Sample	% Inhibition				
	100 ppm	200 ppm	300 ppm	400 ppm	1000 ppm
SSE	49.44 ± 0.15de	49.88 ± 0.1e	50.96 ± 0.07d	54.65 ± 0.07b	66.71 ± 0.04a
SPE	53.46 ± 0.1b	53.98 ± 0.13a	54.36 ± 0.04a	54.71 ± 0.11b	56.24 ± 0.11g
SSW	49.05 ± 0.09f	49.48 ± 0.11f	50.1 ± 0.06f	50.62 ± 0.07g	56.29 ± 0.09g
SPW	49.53 ± 0.09d	50.07 ± 0.04d	50.59 ± 0.13e	51.09 ± 0.04f	58.57 ± 0.03c
SSEW	49.2 ± 0.05ef	53.08 ± 0.07b	54.31 ± 0.14a	55.08 ± 0.02a	63.14 ± 0.07b
SPEW	49.61 ± 0.05d	49.87 ± 0.1e	50.2 ± 0.15f	50.65 ± 0.09g	57.24 ± 0.07d
ESS	52.01 ± 0.07c	53.88 ± 0.07a	54.3 ± 0.11a	54.76 ± 0.09ab	57.28 ± 0.11d
EPS	49.08 ± 0.17f	49.53 ± 0.07f	50.95 ± 0.17d	53.08 ± 0.11d	55.8 ± 0.15h
WSS	49.47 ± 0.15de	49.91 ± 0.09de	50.58 ± 0.15e	51.29 ± 0.16f	56.47 ± 0.09f
WPS	49.6 ± 0.55d	49.87 ± 0.11e	52.01 ± 0.06c	52.49 ± 0.1e	53.11 ± 0.06j
EWSS	52.02 ± 0.07c	52.43 ± 0.17c	52.92 ± 0.05b	53.96 ± 0.14c	54.66 ± 0.04i
EWPS	49.62 ± 0.07d	49.87 ± 0.11e	50.27 ± 0.09f	52.41 ± 0.09e	56.85 ± 0.05e
ESC	42.26 ± 0.00g	43.07 ± 0.03g	44.46 ± 0.06g	48.01 ± 0.7h	49.16 ± 0.00k
EPC	40.81 ± 0.03h	41.38 ± 0.02h	42.56 ± 0.00h	42.95 ± 0.03i	45.27 ± 0.04e
TBHQ	67.63 ± 0.01a				

Values (Mean ± SD, *n* = 3) in the same column with different letters are significantly different (*P* < 0.05).

*Note:* Solvent extracts of skin by ethanol (SSE), water (SSW), and ethanol–water (SSEW). Solvent extracts of pulp by ethanol (SPE), water (SPW) and ethanol–water (SPEW). Ultrasound-assisted extract of ethanol (ESS), water (WSS), and ethanol–water (EWSS). Ultrasound-assisted extract of ethanol (EPS), water (WPS) and ethanol–water (EWPS). Ethanol extract of skin by supercritical CO_2_ method (ESC). Ethanol extract of pulp by the supercritical CO_2_ method (EPC).

### *β*-Carotene bleaching assay

In this assay, *β*-carotene is oxidized by free radicals derived from the oxidation of linoleic acid, which can better simulate the food system compared to DPPH assay (Razali et al. 2012). The auto-oxidation products of linoleic acid attack the double bonds of *β*-carotene and in the absence of an antioxidant the *β*-carotene molecule loses its chromosphere and undergoes rapid discoloration which can be monitored spectrophotometrically (Martinez-Correa et al. 2011). However, in the presence of an antioxidant, *β*-carotene retains its original yellow color by scavenging the free radicals formed in the system (Razali et al. 2012). Effect of loquat fruit skin and pulp extracts on oxidation of *β*-carotene/linoleic acid emulsion is shown in Table3. It was clear that the presence of extracts reduced the oxidation of *β*-carotene. There were significant differences (*P* < 0.05) between the skin and pulp extracts, control (no added antioxidant), and the reference standard (TBHQ). The antioxidant activity of SSEW in levels of 100 and 200 ppm, SSE, SSEW (with no significant difference) in levels 300 and 400 ppm and SSW in level 1000 ppm were higher than other samples. Overall, the antioxidant activity effect of skin extracts on reducing the oxidation of *β*-carotene was higher than the control and pulp extracts, but the best antioxidant power was observed in TBHQ. This suggests that the content of phenolic and tocopherol compounds can play a major role in the antioxidant activity of skin extracts compared to pulp extracts. Results also showed that solvent extraction was more effective on antioxidant activity of loquat extracts compared to other methods. These results concurred with Koba et al. (2007) which examined the antioxidant effect of loquat fruit skin and pulp by the *β*-carotene bleaching assay.

**Table 3:** Antioxidant activity of loquat fruit skin and pulp extracts in a *β*-carotene/linoleate model system.

Sample	% Inhibition				
	100 ppm	200 ppm	300 ppm	400 ppm	1000 ppm
SSE	33.33 ± 2.2c	55.41 ± 5.95b	78.33 ± 3.33a	85.58 ± 3.51ab	64.58 ± 2.08b
SPE	23.01 ± 1.99ef	35.83 ± 5.1cd	45.83 ± 2.08de	68.75 ± 2.76ef	60.41 ± 0.00bcd
SSW	29.08 ± 2.19cd	32.16 ± 1.43d	58.33 ± 5.51b	70.83 ± 3.91e	68.75 ± 3.61a
SPW	27.08 ± 3.6de	33.33 ± 4.17d	50 ± 4.17cd	64.33 ± 3.99f	58 ± 1.66de
SSEW	46.66 ± 5.86b	62.91 ± 2.66a	77.08 ± 4.17a	83.41 ± 2.63bc	62.41 ± 0.97bc
SPEW	28.5 ± 1.64d	37.66 ± 0.71cd	54.41 ± 0.19bc	67.66 ± 4.61ef	54.16 ± 2.39ef
ESS	24.16 ± 2.62def	34.58 ± 1.85d	39.16 ± 1.64f	77.08 ± 5.64d	56.25 ± 2.34de
EPS	22.91 ± 3.4ef	35.8 ± 5.95cd	45.34 ± 2.06de	55.32 ± 4.56g	44.16 ± 3.77g
WSS	22.83 ± 2.11ef	37.5 ± 2.08cd	56.66 ± 2.28b	69.75 ± 1.13ef	58.75 ± 1.92cd
WPS	22.58 ± 3.07ef	34.73 ± 2.58d	43.72 ± 3.44ef	52.04 ± 2.82g	40.27 ± 1.34g
EWSS	23.08 ± 2.18ef	36.6 ± 3.57cd	43.33 ± 2.32ef	79.16 ± 2.08cd	52.08 ± 2.08f
EWPS	27.16 ± 3.45de	36.58 ± 4.14cd	55.33 ± 2.56b	65.7 ± 0.77ef	60.43 ± 5.17bcd
ESC	21.74 ± 0.01f	41.66 ± 0.12c	55.69 ± 0.02b	64.73 ± 0.02f	51.24 ± 0.02f
EPC	20.04 ± 0.03f	34.34 ± 0.02d	45.65 ± 0.04de	50.47 ± 0.04g	43.47 ± 0.02g
TBHQ	89.58 ± 0.04a				

Values (Mean ± SD, *n* = 3) in the same column with different letters are significantly different (*P* < 0.05).

*Note:* Solvent extracts of skin by ethanol (SSE), water (SSW), and ethanol–water (SSEW). Solvent extracts of pulp by ethanol (SPE), water (SPW), and ethanol–water (SPEW). Ultrasound-assisted extract of ethanol (ESS), water (WSS), and ethanol–water (EWSS). Ultrasound-assisted extract of ethanol (EPS), water (WPS), and ethanol–water (EWPS). Ethanol extract of skin by supercritical CO_2_ method (ESC). Ethanol extract of pulp by the supercritical CO_2_ method (EPC).

### Rancimat analysis

The Rancimat analysis was performed at 120°C and the induction period (h) was evaluated for soybean oils with or without loquat fruit skin and pulp extracts at 400 and 1000 ppm. Our results showed both skin and pulp extracts had a strong antioxidant activity in soybean oil. As it has been shown in Table4, the presence of extracts retarded the oxidation of soybean oil, so that the lowest thermal stability in oils treated with extracts was for the EPC sample (3.8 h), which was higher compared to the control oil (3.32 h). On the other hand, the highest thermal stability in oils containing loquat fruit skin and pulp extracts was for SSEW at 400 and 1000 ppm (4.69 and 4.49 h), but the best protection effect was observed in oil containing 100 ppm of TBHQ. Therefore, results also showed that the skin extracts was more effective in prevention of soybean oil oxidation compared to pulp extracts. These results concurred with Sun and Ho (2005) and Aladedunye and Matthäus (2014) that reported the induction period of oils containing buckwheat, rowanberry, and crabapple extracts was higher than control oil, but the most antioxidant effect was in TBHQ. Moreover, the findings of this assay showed that the samples extracted by the solvent extraction method exhibited strong antioxidant activity under the Rancimat test conditions.

**Table 4:** Antioxidant activity of loquat fruit skin and pulp extracts analyzed by the Rancimat method.

Sample	Induction time (h)	
	400 ppm	1000 ppm
SSE	4.69 ± 0.06b	4.49 ± 0.06b
SPE	4.58 ± 0.03c	4.42 ± 0.04b
SSW	4.3 ± 0.06fg	3.91 ± 0.04f
SPW	4.26 ± 0.04gh	4.2 ± 0.05cde
SSEW	4.78 ± 0.03b	4.66 ± 0.05a
SPEW	4.46 ± 0.06de	4.4 ± 0.06b
ESS	4.32 ± 0.07fg	4.09 ± 0.06e
EPS	4.03 ± 0.05i	3.87 ± 0.1f
WSS	4.39 ± 0.03ef	4.11 ± 0.05de
WPS	4.18 ± 0.04h	4.11 ± 0.05de
EWSS	4.51 ± 0.05cd	4.25 ± 0.07c
EWPS	4.33 ± 0.06fg	4.22 ± 0.05cd
ESC	3.94 ± 0.08i	3.92 ± 0.04f
EPC	3.81 ± 0.06j	3.66 ± 0.07g
TBHQ	6.48 ± 0.05a	
Control	3.22 ± 0.03k	

Values (Mean ± SD, *n* = 3) in the same column with different letters are significantly different (*P* < 0.05).

*Note:* Solvent extracts of skin by ethanol (SSE), water (SSW), and ethanol–water (SSEW). Solvent extracts of pulp by ethanol (SPE), water (SPW), and ethanol–water (SPEW). Ultrasound-assisted extract of ethanol (ESS), water (WSS), and ethanol–water (EWSS). Ultrasound-assisted extract of ethanol (EPS), water (WPS), and ethanol–water (EWPS). Ethanol extract of skin by supercritical CO_2_ method (ESC). Ethanol extract of pulp by the supercritical CO_2_ method (EPC).

## Conclusions

Generally, the results of the present study indicated that the solvent extraction was more effective in antioxidant activity of loquat fruit extracts in the DPPH˙ free radical assay, *β*-carotene bleaching, and the Rancimat methods. So, it is suggested that the best method for the extraction of phenolic and tocopherol compounds from loquat fruit skin and pulp is by the solvent extraction method using the ethanol–water mixture. Furthermore, antioxidant activities of loquat skin extracts were higher than the pulp extracts. The high antioxidant activity of the skin extracts appeared to be attributed to its high tocopherol and phenolics content. Therefore, loquat fruit skin and pulp extracts could be used as natural antioxidants to enhance the antioxidant properties of functional food.

## Conflict of Interest

None declared.
